# Supplementary material for: Automated cell annotation in multi-cell images using an improved CRF_ID algorithm
Source: bioRxiv. 2023 Nov 23:2023.06.07.543949. Originally published 2023 Jun 8. Preprint. [Version 2] doi: 10.1101/2023.06.07.543949 (PMC10274780; doi:10.1101/2023.06.07.543949)
Supplement: Supplement 1 [file NIHPP2023.06.07.543949v2-supplement-1.pdf]

bioRxiv preprint doi: https://doi.org/10.1101/2023.06.07.543949; this version posted November 23, 2023. The copyright holder for this preprint (which was not certified by peer review) is the author/funder, who has granted bioRxiv a license to display the preprint in perpetuity. It is made available under aCC-BY 4.0 International license.

Supplementary Figures

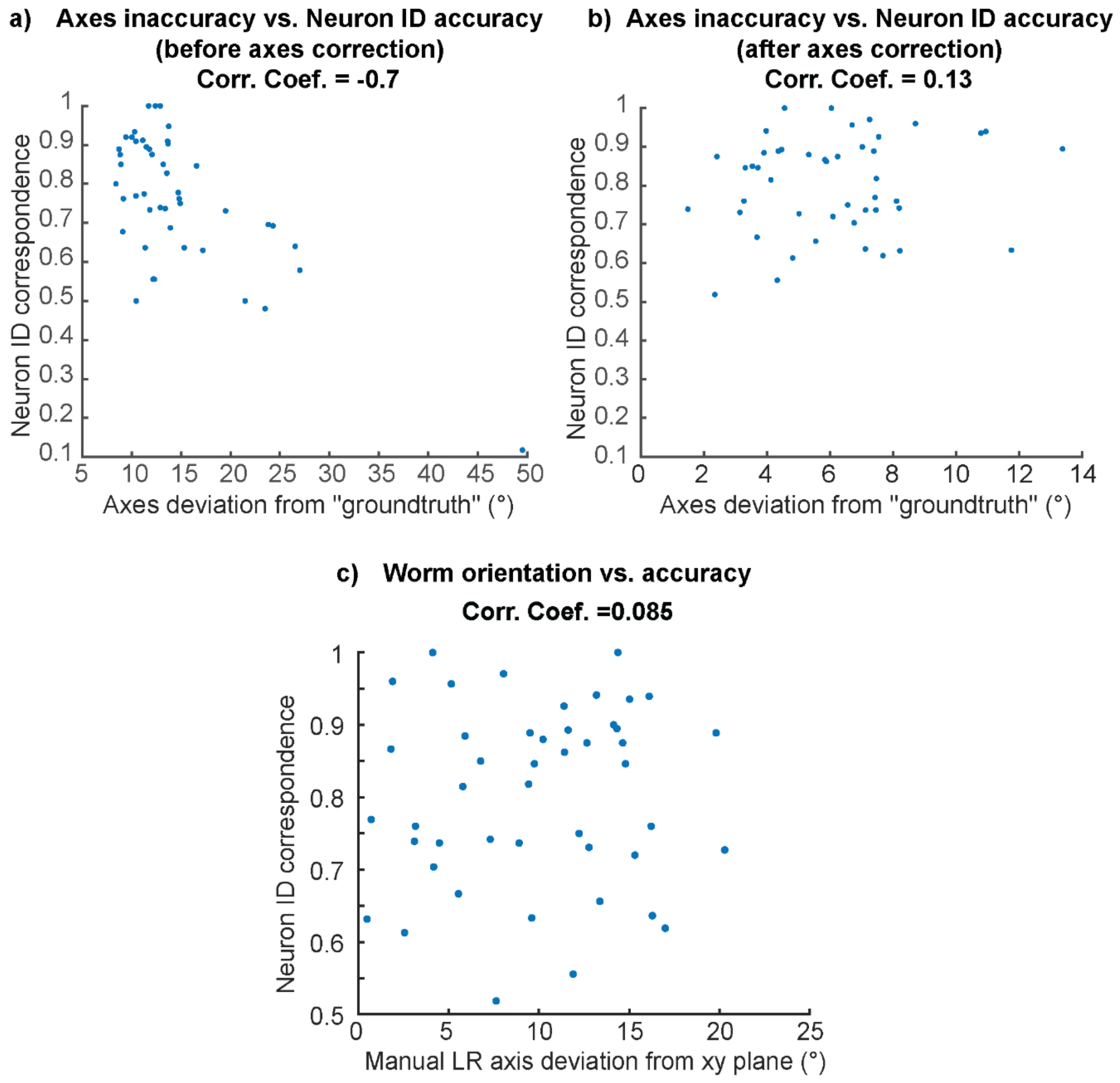

Figure 2- figure supplement. Neuron ID accuracy no longer depends on the axes inaccuracy after axes correction. a) High negative correlation between axes inaccuracy and neuron ID accuracy before axes correction. b) No correlation between axes inaccuracy and neuron ID accuracy after axes correction. c). No correlation between worm orientation and neuron ID accuracy.

bioRxiv preprint doi: https://doi.org/10.1101/2023.06.07.543949; this version posted November 23, 2023. The copyright holder for this preprint (which was not certified by peer review) is the author/funder, who has granted bioRxiv a license to display the preprint in perpetuity. It is made available under aCC-BY 4.0 International license.

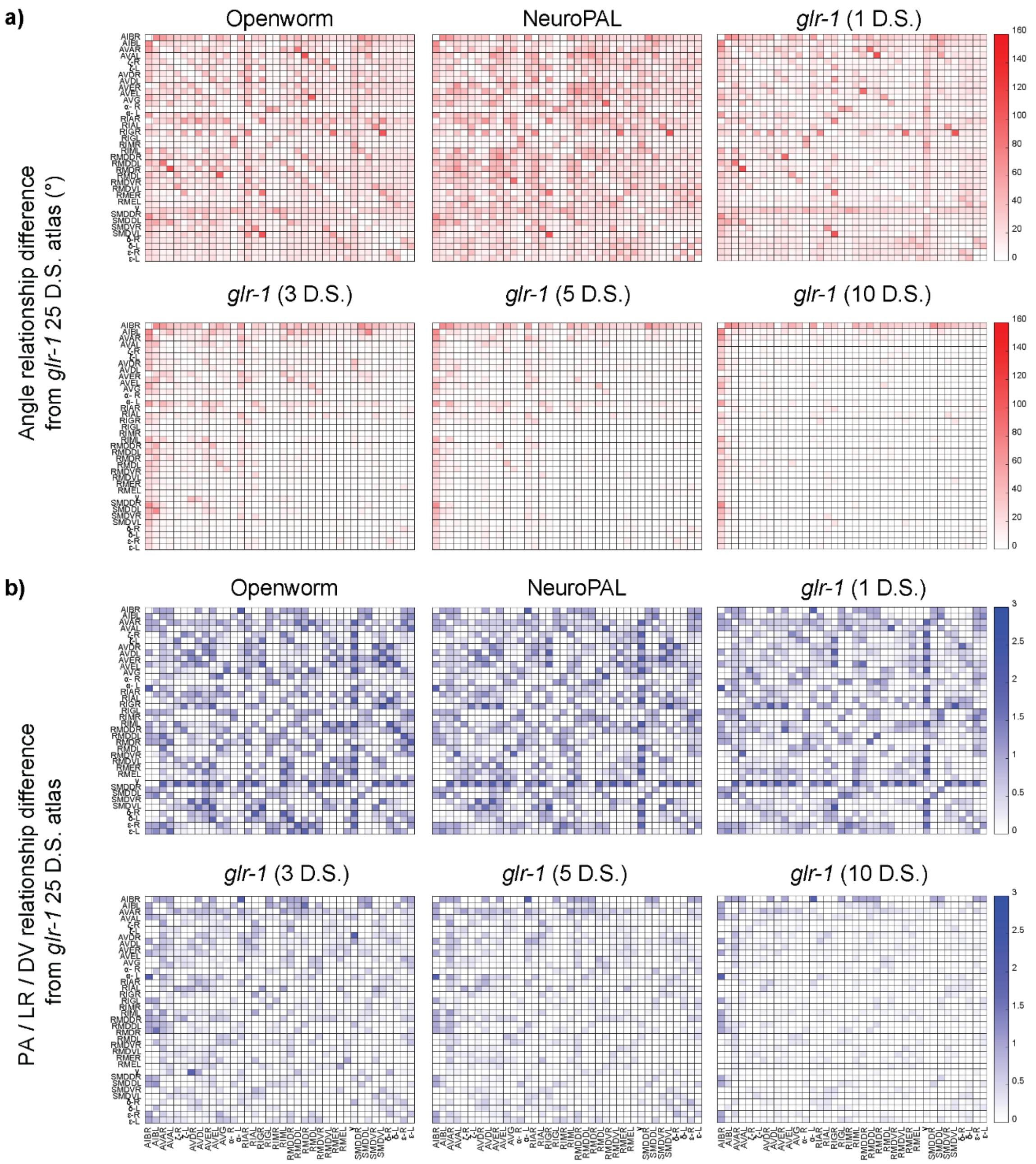

Figure 3- figure supplement 1. A more detailed visual representation of the difference of each atlas from the best available atlas (*glr-1* from 25 datasets (D.S.)). a) differences in angular relationships. The red color intensity indicates the angle differences of neuron pair vectors in the particular atlas and those in the best available atlas. b) differences in PA/LR/DV relationships. The blue color intensity (ranging from 0 to 3) indicates the summed absolute differences in the three pairwise relationships between the atlas and the best available atlas.

bioRxiv preprint doi: https://doi.org/10.1101/2023.06.07.543949; this version posted November 23, 2023. The copyright holder for this preprint (which was not certified by peer review) is the author/funder, who has granted bioRxiv a license to display the preprint in perpetuity. It is made available under aCC-BY 4.0 International license.

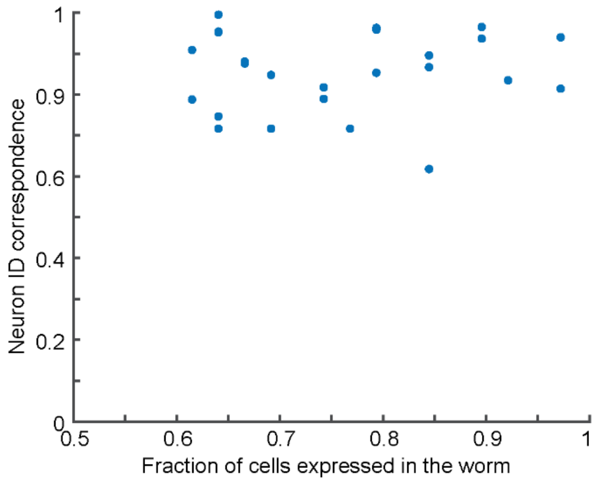

Figure 3- figure supplement 2. No correlation between the degree of mosaicism (fraction of cells expressed in the worm) and neuron ID correspondence.

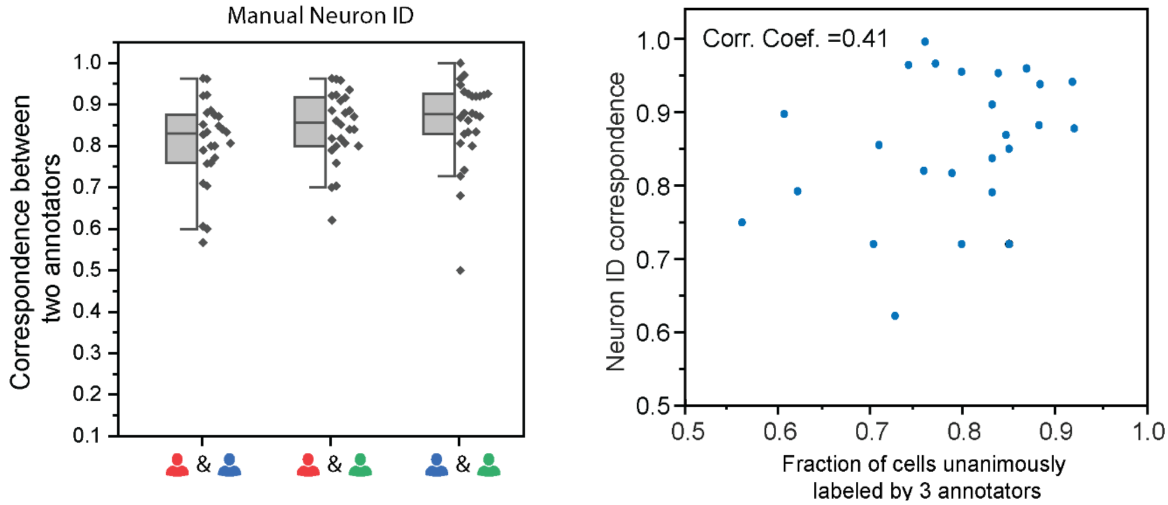

Figure 4- figure supplement. a) correspondence between any two among the three annotators. b) Slight correlation between the fraction of cells unanimously labeled by 3 annotators and neuron ID correspondence.

bioRxiv preprint doi: https://doi.org/10.1101/2023.06.07.543949; this version posted November 23, 2023. The copyright holder for this preprint (which was not certified by peer review) is the author/funder, who has granted bioRxiv a license to display the preprint in perpetuity. It is made available under aCC-BY 4.0 International license.

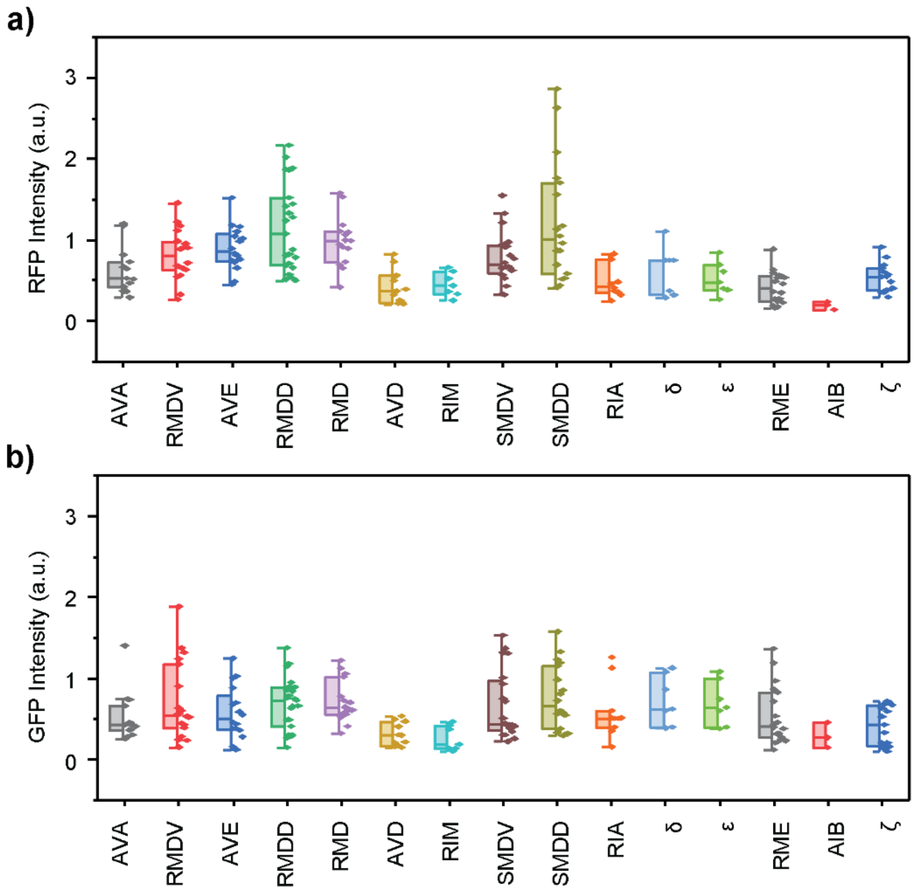

Figure 5- figure supplement 1. Neuron-specific expressions of the *glr-1* gene for neurons on the "dim" side of the specimen. These left/right paired neurons are on the side of the *C. elegans* farther away from the objective. a) Extrachromosomal transgene expression. b) Integrated transgene expression.

bioRxiv preprint doi: https://doi.org/10.1101/2023.06.07.543949; this version posted November 23, 2023. The copyright holder for this preprint (which was not certified by peer review) is the author/funder, who has granted bioRxiv a license to display the preprint in perpetuity. It is made available under aCC-BY 4.0 International license.

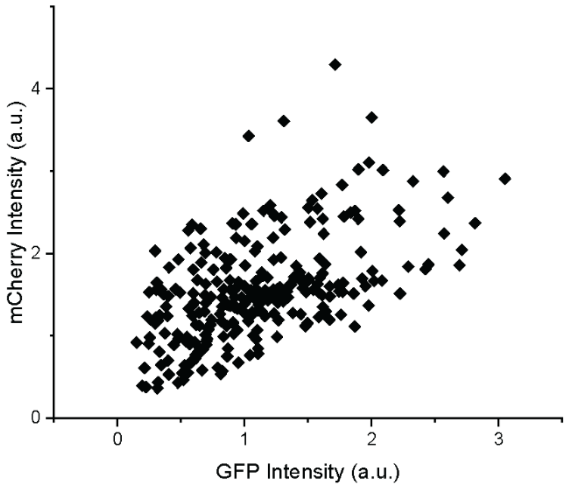

Figure 5- figure supplement 2. High correlation between extrachromosomal (mCherry) and integrated (GFP) transgene expressions. Each data point indicates the GFP and mCherry (RFP) intensities of a single neuron.
