# Supplementary material for: Automated cell annotation in multi-cell images using an improved CRF_ID algorithm
Source: bioRxiv. 2023 Jun 8:2023.06.07.543949. Preprint. [Version 1] doi: 10.1101/2023.06.07.543949 (PMC10274780; doi:10.1101/2023.06.07.543949)
Supplement: Supplementary file 1 [file NIHPP2023.06.07.543949V1-supplement-1.pdf]

bioRxiv preprint doi: https://doi.org/10.1101/2023.06.07.543949; this version posted June 8, 2023. The copyright holder for this preprint (which was not certified by peer review) is the author/funder, who has granted bioRxiv a license to display the preprint in perpetuity. It is made available under aCC-BY 4.0 International license.

Supplementary Figures

bioRxiv preprint doi: https://doi.org/10.1101/2023.06.07.543949; this version posted June 8, 2023. The copyright holder for this preprint (which was not certified by peer review) is the author/funder, who has granted bioRxiv a license to display the preprint in perpetuity. It is made available under aCC-BY 4.0 International license.

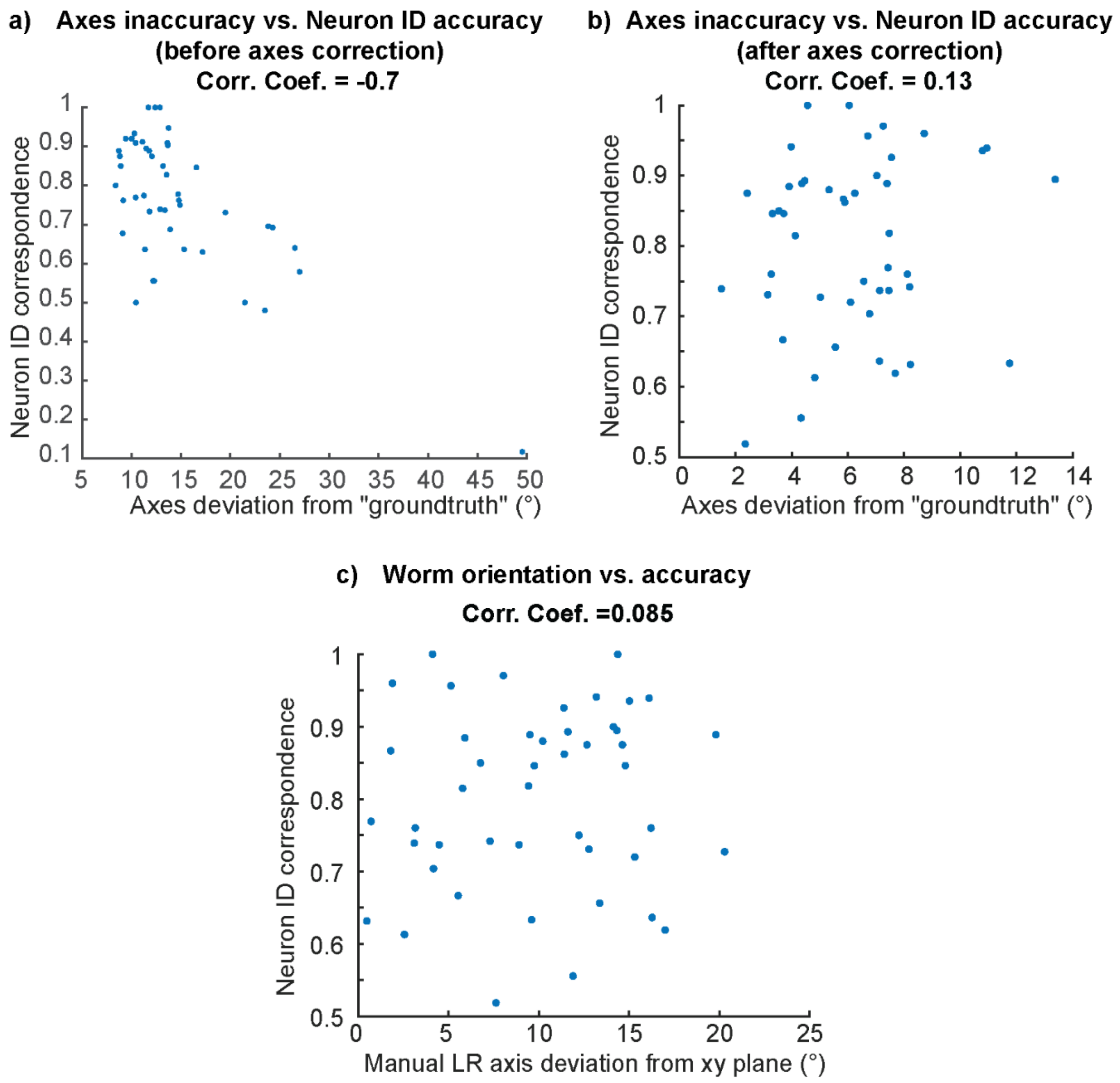

Figure 2- figure supplement. Neuron ID accuracy no longer depends on the axes inaccuracy after axes correction. a) High negative correlation between axes inaccuracy and neuron ID accuracy before axes correction. b) No correlation between axes inaccuracy and neuron ID accuracy after axes correction. c). No correlation between worm orientation and neuron ID accuracy.

bioRxiv preprint doi: https://doi.org/10.1101/2023.06.07.543949; this version posted June 8, 2023. The copyright holder for this preprint (which was not certified by peer review) is the author/funder, who has granted bioRxiv a license to display the preprint in perpetuity. It is made available under aCC-BY 4.0 International license.

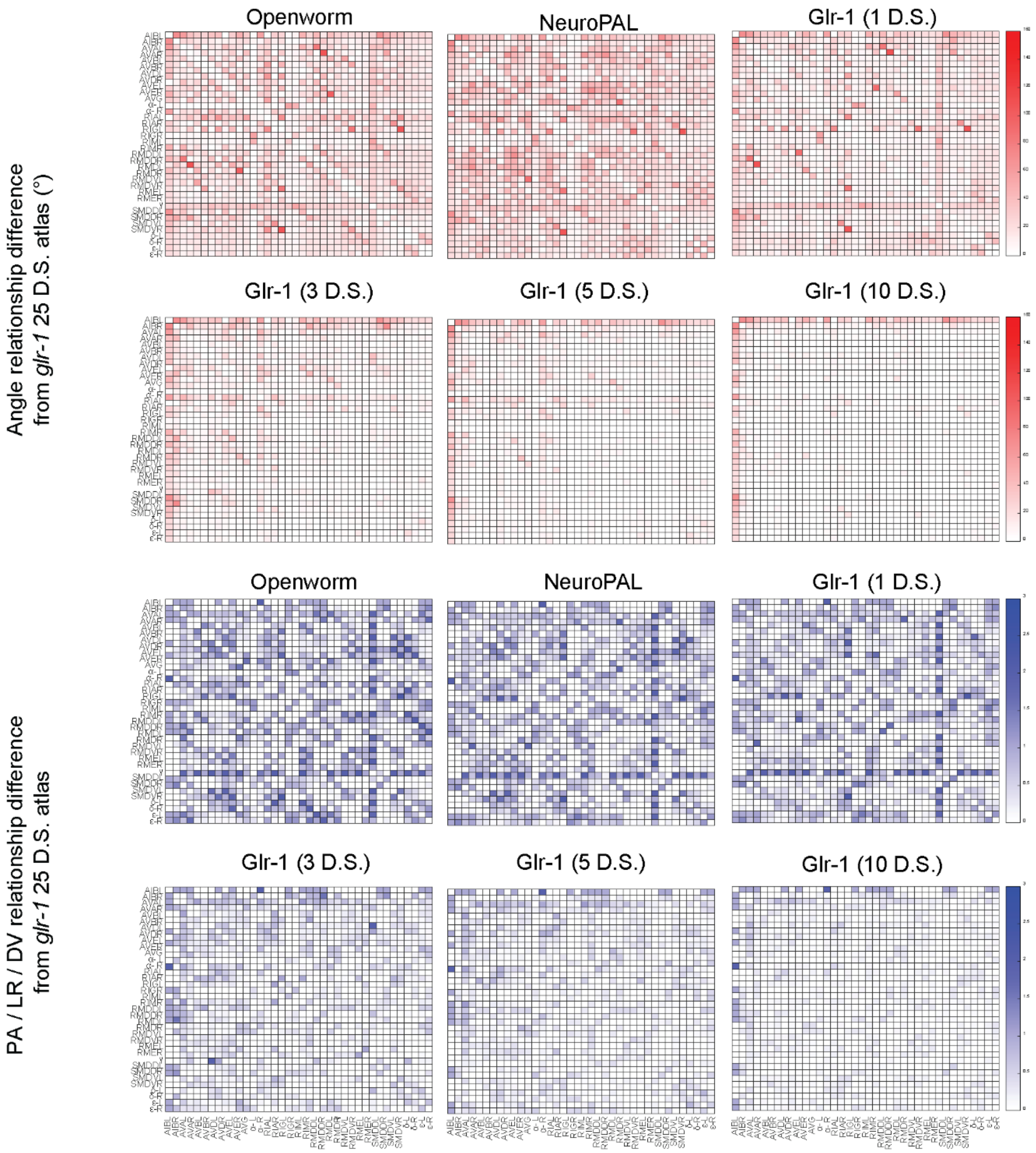

Figure 3- figure supplement. A more detailed visual representation of the difference of each atlas from the best available atlas (*glr-1* from 25 datasets). a) differences in angular relationships.b) differences in PA/LR/DV relationships.

bioRxiv preprint doi: https://doi.org/10.1101/2023.06.07.543949; this version posted June 8, 2023. The copyright holder for this preprint (which was not certified by peer review) is the author/funder, who has granted bioRxiv a license to display the preprint in perpetuity. It is made available under aCC-BY 4.0 International license.

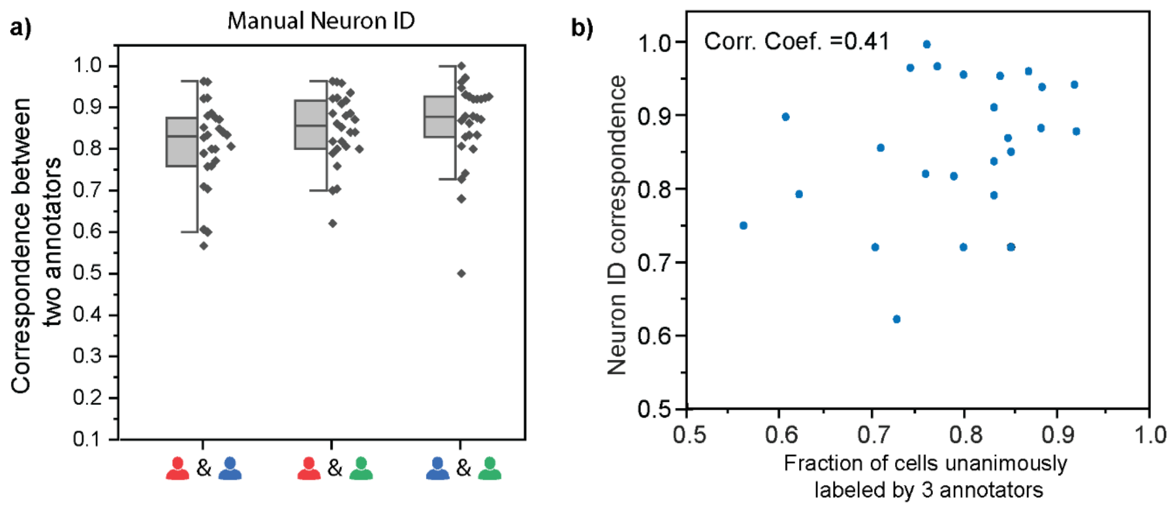

Figure 4- figure supplement. a) correspondence between any two among the three annotators. b) Slight correlation between the fraction of cells unanimously labeled by 3 annotators and neuron ID correspondence.

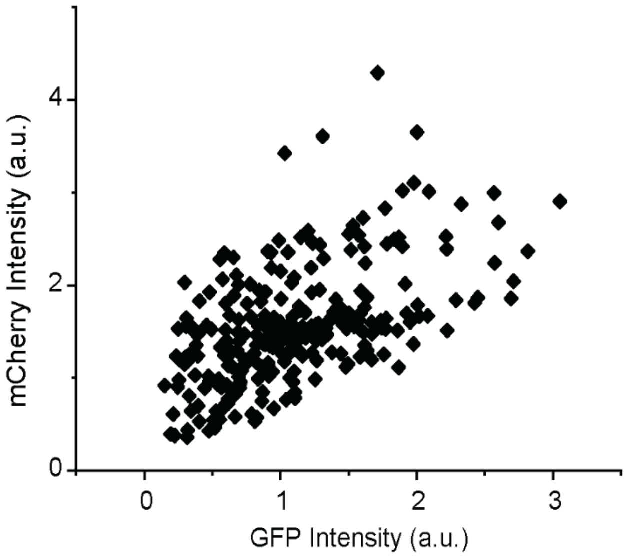

Figure 5- figure supplement 2. High correlation between extrachromosomal (mCherry) and integrated (GFP) transgene expressions.
